# Logging intensity alters tree species composition and wood density, but not tree diversity, in lowland forests in Vietnam

**DOI:** 10.1007/s10531-026-03283-2

**Published:** 2026-03-14

**Authors:** Suzanne M. Stas, Ervan Rutishauser, Tue Van Ha, Tinh Cong Le, Hieu Dang Tran, Trai Trong Le, Benedict D. Spracklen, Douglas Sheil, Marijke van Kuijk, Oliver L. Phillips, Dominick V. Spracklen

**Affiliations:** 1 System Earth Science, Maastricht University, PO Box 616, Maastricht, 6200 MD The Netherlands; 2 School of Earth and Environment, University of Leeds, Leeds, LS2 9JT UK; 3 InfoFlora, Conservatory and Botanical Gardens, PO Box 71, Chambésy-Genève, CH-1292 Switzerland; 4 Forest Inventory and Planning Institute, Vinh Quynh, Thanh Tri, Hanoi, Vietnam; 5 Viet Nature Conservation Centre, No. 6 Dinh Le Street, PO Box 89, Hanoi, Vietnam; 6 Forest Ecology and Forest Management Group, Wageningen University & Research, PO Box 47, Wageningen, 6700 AA The Netherlands; 7 Wildlife Ecology and Nature Restoration, Utrecht University, PO Box 80056, Utrecht, 3508 TB The Netherlands; 8 School of Geography, University of Leeds, Leeds, LS2 9JT UK

**Keywords:** Forest degradation, Tree census, Biodiversity, Timber species, Carbon storage, Southeast Asia

## Abstract

Tropical forests host considerable biodiversity but face degradation from timber extraction (“logging”). We examined how logging intensity affected tree diversity, species composition, community wood density and availability of timber species in a lowland forest in north-central Vietnam. We measured and identified trees in 18 quarter-hectare plots that vary in historical logging intensity. Tree diversity showed no significant relationships with logging intensity. However, species composition differed, with each logging intensity class having distinct abundant tree species. Community wood density decreased significantly with logging intensity, being 9% lower in heavily than in lightly logged forests. Timber species were scarce overall, and individuals of harvestable size were most common in lightly logged forests. These results show that while tree diversity can persist after logging, heavy logging shifts community composition towards lighter-wood species and depletes valuable timber trees, reducing both carbon storage and timber potential. Reducing logging intensities is therefore critical for maintaining and increasing community wood density, carbon stocks and sustainable timber supplies in these forests in north-central Vietnam.

**Supplementary Information:**

The online version contains supplementary material available at 10.1007/s10531-026-03283-2.

## Introduction

Tropical forests support many of the world’s species and provide additional ecosystem services including carbon storage, local climate regulation and mitigation of both floods and droughts (Pan et al. 2013; Ellison et al. 2017; Mitchard 2018). Yet many are seriously threatened by forest degradation, primarily driven by timber extraction (“logging”) (Hosonuma et al. 2012; Pearson et al. 2014). Selective logging typically targets specific species and size classes and alters biotic and abiotic processes that regulate resource availability (Ramírez-Marcial 2003; Clark and Covey 2012). This in turn affects plant growth, recruitment and mortality and interactions among species (Putz et al. 2000; Ramírez-Marcial 2003).

A meta-analysis showed that selectively logged tropical forests can retain high biodiversity values, despite the damage caused by logging (Putz et al. 2012). Another meta-analysis that conducted pairwise comparisons of biodiversity values in human-disturbed and primary tropical forests found that selectively logged forests had a significant reduction in tree species richness (Clark and Covey 2012). In contrast, many individual studies across the tropics (Borneo, China and Central African Republic) found that logged forests support higher or at least similar tree diversity compared to unlogged forests (Cannon et al. 1998; Sheil et al. 1999; Hall et al. 2003; Berry et al. 2008, 2010; Ding et al. 2012; Imai et al. 2012). Post-logging, most species usually persist at reduced densities and additionally the increased heterogeneity of forest microhabitats creates opportunities for the establishment of immigrant species, leading often to higher diversity in disturbed areas (Sheil et al. 1999). Especially logging intensity is an important factor in determining differences in tree species richness in logged tropical forests (Martin et al. 2015). Usually tree species richness increases at low logging intensities but decreases at higher intensities (Martin et al. 2015), which is consistent with the intermediate disturbance hypothesis (IDH) that predicts highest local species diversity at an intermediate level of disturbance (Connell 1978; Sheil and Burslem 2003; Bongers et al. 2009).

Change in species richness per se does not provide information about the conservation value of individual species (Sheil et al. 1999), therefore it is also important to assess the impacts on species composition. Logging can affect tree species composition by changing the abundance and distribution of species (Putz et al. 2000). Evidence on the effects of logging on tree species composition is mixed: some studies reported similar compositions between logged and unlogged forests (Central African Republic and China; Hall et al. 2003; Ding et al. 2012), while others showed differences between logged and unlogged forests (Borneo; Berry et al. 2008) and between logging intensities (Vietnam; Hoang et al. 2011). Generally, more early successional tree species and species of low conservation value are observed in (heavily) logged or disturbed forests, while vulnerable species and valuable timber species are most common in lightly logged or undisturbed forests (meta-analysis in tropical and temperate forests and individual studies in Vietnam, Ghana and Bolivia; Bongers et al. 2009; Carreño-Rocabado et al. 2012; Clark and Covey 2012; Hoang et al. 2011).

Changes in species composition can result in shifts in community wood density. Previous work in Borneo found that average wood densities in forests decreased with disturbance levels (Slik et al. 2008) and that logging changed the fraction of softwood stems across diameter classes (Verburg and Van Eijk-Bos 2003). First harvests in old-growth tropical forests are often selective, with high-density timber species being targeted, while later harvesting may need to make do by removing lower-density species. Post-logging, timber trees are generally more abundant in lighter logged forests than in heavy logged sites (Vietnam; Hoang et al. 2008, 2011). Further, large logging gaps can be rapidly colonized by pioneer species and lianas, which strongly limit the regeneration of timber species after logging (Bolivia and Borneo; Fredericksen and Mostacedo 2000; Sist and Nguyen-The 2002).

Most previous studies on the impacts of logging on tree diversity and composition in Southeast Asian forests were conducted in Borneo, while relatively few studies have focused on mainland Southeast Asia. Vietnam has high levels of species richness and endemism of vascular plants, partly due to Southeast Asia’s complex geological and climatic past, Vietnam’s wide range of latitudes covering a transition from subtropical to tropical zones and the relatively hilly and mountainous topography in Vietnam (Sterling and Hurley 2005; MONRE 2014). Vast areas of forests in Vietnam experienced intense state and illegal logging over the past century (McElwee 2004). Various studies assessed the tree diversity and composition in logged forests in Vietnam (Tran et al. 2005; Millet and Truong 2011; Do et al. 2019; Hai and Quang 2019), but only one study (Hoang et al. 2011) considered variations in logging intensity.

Khe Nuoc Trong forest (KNT) in north-central Vietnam is one of the last remaining extensive lowland forests in the Annamite Mountains and harbours species of exceptional conservation value (Department for Agriculture and Rural Development 2010). KNT experienced various levels of state logging in the past and ongoing illegal logging (Stas et al. 2020). Previous work showed that variations in above-ground carbon (AGC) stocks across KNT could be explained by differences in logging intensities (Stas et al. 2020). To improve biodiversity conservation, it is important to understand the impacts of historical logging intensities on floristics in KNT. Here, we investigate the impacts of historical logging intensities on tree diversity, species composition, community wood density and availability of timber species in KNT.

## Materials and methods

### Site description

**Fig. 1:**
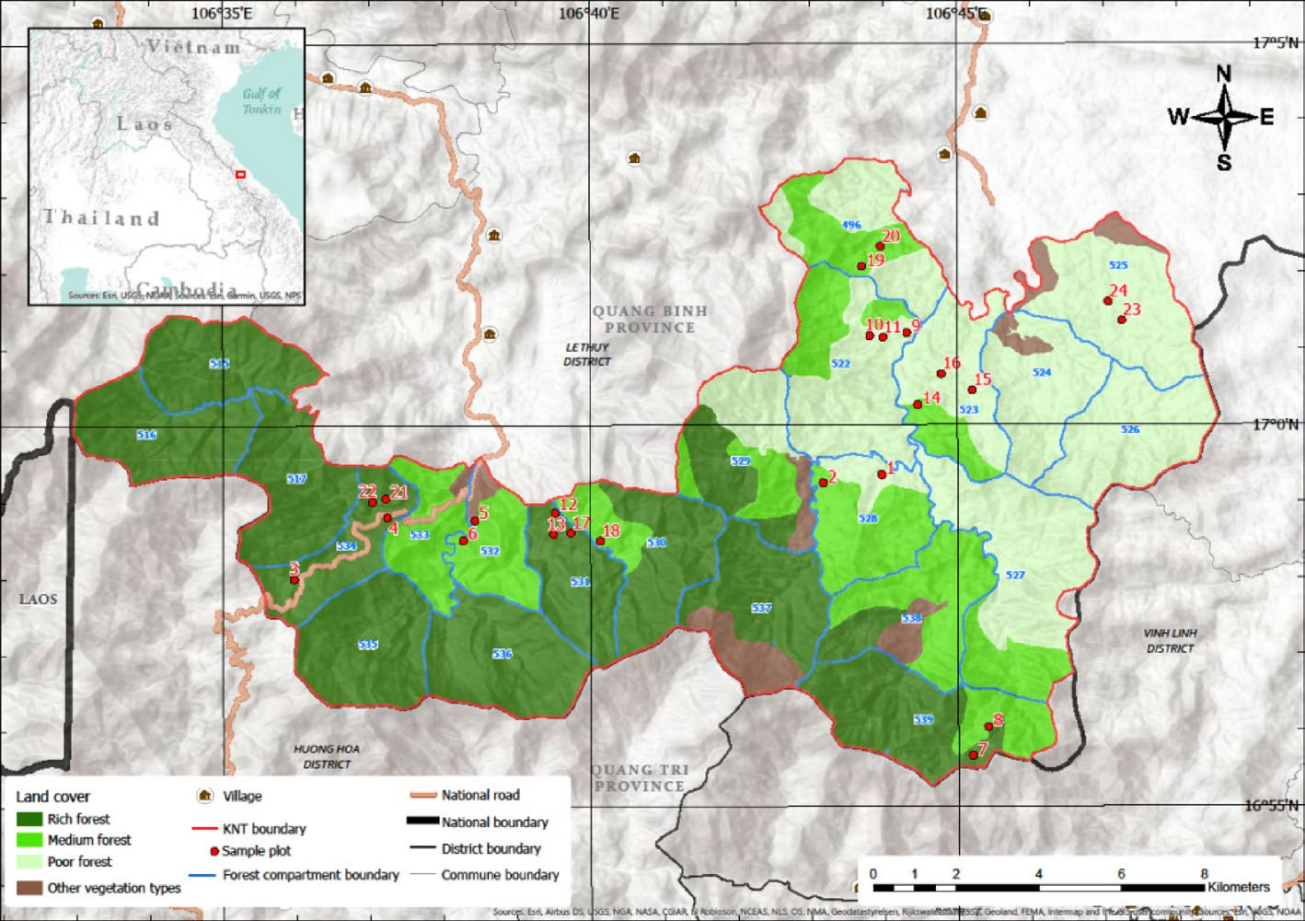
Vegetation map of KNT with the locations of the 24 sample plots (land cover from FIPI (2010))

KNT is an evergreen tropical forest of approximately 22,000 ha (~ 20,000 ha at the time of sampling) located in Le Thuy District in Quang Binh Province in north-central Vietnam (Fig. 1). Elevation in KNT ranges from 120 to 1220 m, with the majority of the area (90%) < 700 m altitude on hilly terrain (Department for Agriculture and Rural Development 2010). The area has a tropical monsoon climate with hot summers and relatively cold winters and storms from June to September (Mahood and Hung 2008; pers. comm. Trai Trong Le, 2019). Total annual rainfall varies between 2400 and 2800 mm, with a drier period for a few months per year (Mahood and Hung 2008).

KNT is a key biodiversity area and was officially established as a Nature Reserve in 2020, which is Vietnam’s highest governmental protection status. KNT was selectively logged by the state between 1982 and 2007. Since 2007 logging has been forbidden, but widespread illegal logging still occurs (Ngo et al. 2020). Trees are harvested both for local use and for sale (Ngo et al. 2020). At least 14 tree species are extracted for timber in KNT, of which *Erythrophleum fordii*, *Sindora siamensis* and *Sindora tonkinensis* are the most valuable timber species (pers. comm. Viet Nature Conservation Centre, 2018). Local communities also rely on the forest for firewood, honey and other non-timber forest products as well as fishing and hunting forest wildlife (Ngo et al. 2020). The forest contains areas with various levels of forest degradation, from forests that have experienced very little logging disturbance to heavily degraded forests. Vietnam experiences 5–6 tropical cyclones a year (Lap 2019) and KNT is regularly impacted by these cyclones, which damage the forest (Stas et al. 2022). KNT has not been affected by the defoliants during the Vietnam War. More site details are described in Stas et al. (2020).

### Field measurements

We have established 24 plots of 0.25 ha each in KNT's lowland forests (< 700 m elevation) between April 2016 and June 2017 (Stas et al. 2020; Fig. 1). Here we focus our analyses on plots 1–18 established in 2016, of which trees were identified by the same botanist. Vietnamese forests have been classified into poor, medium and rich forests, based on timber reserves of standing trees (stocking classes; MARD 2009). Differences in above-ground biomass between poor, medium and rich forests in KNT are a result of historical timber harvesting (Ngo et al. 2020; Stas et al. 2020). Historical logging intensity was assessed through satellite remote sensing as described in the next section. These three stocking classes cover similar percentages of area in the lowlands of KNT (Birdlife International n.d.), therefore sample plots were equally and spatially stratified over poor, medium and rich forests (*n* = 6 per stocking class).

Measurements in the plots were conducted according to the RAINFOR and GEM protocols (Marthews et al. 2014; Phillips et al. 2016; http://www.rainfor.org/en/manuals). Living woody stems ≥ 10 cm diameter at breast height (dbh; i.e. diameter at 130 cm height or above buttresses or stem deformities) were tagged and their dbh measured (Stas et al. 2020). A botanist identified each stem with Vietnamese and scientific species names in the field and, when needed, collected botanical vouchers for further identification in the herbarium of the Forest Inventory and Planning Institute (FIPI) in Hanoi, Vietnam. The dbh and species of stems 5–10 cm dbh were measured and identified in a belt transect of 4 m x 50 m, running through the plot centre (Stas et al. 2020).

### Logging assessment

We identified logged stumps ≥ 10 cm diameter in the plots. Wood typically decays rapidly in most tropical forests (Baker et al. 2007), suggesting that evidence of historical logging (stumps) could rapidly vanish in the field. While recent logging intensities can be assessed by identifying stumps in the field, additional data are needed to assess historical logging activities where resulting logged stumps have already decayed and can no longer be identified in the field. Because no accurate records of historical logging were available for our sites, we estimated historical logging intensities using remote sensing analysis and validated these data with participatory mapping as described in Stas et al. (2020). Forest canopy disturbances were identified on Landsat 4/5, 7 and 8 images between 1988 and 2015. We identified disturbance as pixels exhibiting a reduction in the Normalized Burn Ratio (NBR), with two consecutive images showing NBR < 0.5. The disturbance density, a proxy of logging intensity per plot, was estimated by summing the number of disturbed pixels from 1988 to 2015 within 1000 m of each plot centre, with disturbed pixels within 500 m weighted by a factor 2. To confirm logging levels at the sites, participatory mapping with commune and village leaders and households was performed within two villages in Kim Thuy commune (Le Thuy District, Quang Binh Province). For this, maps of KNT with key landmarks were discussed among participants and each area was allocated a logging classification (light, medium, heavy) and a period when the majority of the logging occurred. The forest plots were then allocated a logging classification based on the map. More details and the rationale behind this approach on assessing historical logging intensities are described in Stas et al. (2020).

### Data analysis

#### Logging assessment

Plots were ordered by number of disturbances as identified on the Landsat images and divided over three logging intensity classes (*n* = 6 plots per class): light, medium and heavy. There was good agreement between these classifications from the remote sensing analysis and participatory mapping (Stas et al. 2020). In the analyses we used the logging intensities calculated from the Landsat analysis, when possible the continuous data or otherwise the derived three logging intensity classes.

#### Tree diversity

For all analyses, the number of stems and species ≥ 5 cm dbh were counted per plot, meaning a summation of the number of stems ≥ 10 cm dbh in the 0.25 ha plot and the number of stems 5–10 cm dbh in the 0.02 ha belt transect (without scaling area sizes). All analyses were conducted using R (version 3.5.3) (R Core Team 2019). Taxonomic names were checked and corrected using the Taxonomic Name Resolution Service (Boyle et al. 2013), incorporated in the *BIOMASS* package (Réjou-Méchain et al. 2017). A rarefaction curve was constructed using the function “specaccum” in the *vegan* package (Oksanen et al. 2019). Function “specpool” in the *vegan* package, selecting the first order jackknife, was used to estimate the number of unobserved species in the area and adding them to the observed species richness in the plots (Oksanen et al. 2019). Various diversity metrics were calculated for each plot, i.e. the Shannon index, Simpson’s index, Fisher’s α, species richness (number of species) and evenness, all in the *vegan* package (Oksanen et al. 2019). As diversity typically increases with the number of stems, all diversity metrics were calculated based on the lowest number of total stems in the plots. We calculated mean diversity metrics from 1000 permutations and in each run individuals were randomly selected with replacements, following the same approach as Wunderle et al. (2006). We used ANOVA to test whether the diversity metrics differed between logging intensity classes. Generalized additive models, fitted with the function ”gam” in *mgcv* package (Wood 2011), were used for the relationship between the rarefied Shannon index and logging intensity and between the rarefied species richness and logging intensity.

#### Tree species composition

Non-metric multidimensional scaling (NMDS) using the “metaMDS” function in the *vegan* package (Bray-Curtis Dissimilarity index, 3 dimensions) visualized plot dissimilarity in terms of tree species composition (Oksanen et al. 2019). We used relative species abundance, using the “decostand” function, and created a distance matrix, using the “vegdist“ function with method Bray-Curtis, both in the package *vegan* (Oksanen et al. 2019). Further, we identified the five most abundant tree species for each logging intensity class.

#### Wood density and availability of timber species

Wood densities were extracted from the Global Wood Density Database (Chave et al. 2009; Zanne et al. 2009), incorporated in the *BIOMASS* package (Réjou-Méchain et al. 2017). If the species was present in the database, the global species-level wood density average was assigned. Following standard methods, the wood densities of missing species were attributed by, in subsequent order, the global genus- or plot-level average (Baker et al. 2004; Chave et al. 2006; Slik 2006). Community wood density of stems ≥ 5 cm dbh was averaged by plot and an ANOVA was used to test whether wood density differed with logging intensity.

We assessed the abundance of timber species in the plots, both below and above harvestable diameter size. Official selective logging regulations in Vietnam have been revised multiple times throughout the years and the minimum cutting diameter has varied through time, for different type of wood species and with region, but has been as low as 30 cm diameter (Nam 2017). In our analyses we use a minimum harvest size of 30 cm dbh.

## Results

### Tree diversity

**Fig. 2:**
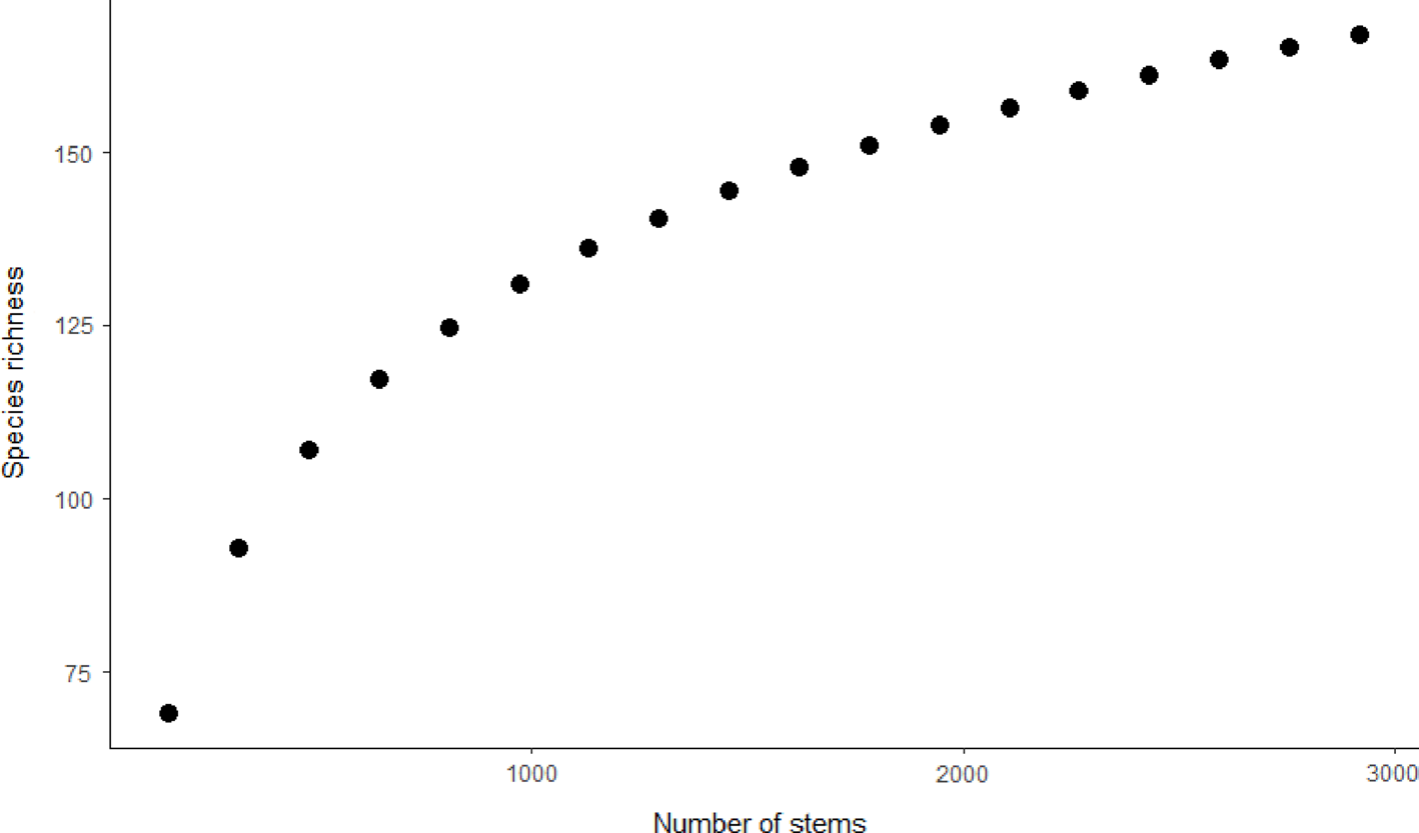
Rarefaction curve for stems ≥ 5 cm dbh for all 18 plots grouped. The number of stems added in each step is equal to the average number of stems per plot, derived from the 18 plots

For the 18 plots combined, 83% of trees were identified to species level and 17% to genus, with < 1% to family or unidentified (see Supplementary Material 1 for proportional frequencies of the levels of tree identifications for each plot). In total, the 2919 stems ≥ 5 cm dbh in the 18 plots represented 167 species (Fig. 2), which is 78% of the estimated total species present in the area (215 species; estimated using the first order jackknife).

The total number of stems ≥ 5 cm dbh increased with historical logging intensity: summing stems in the plots per logging intensity class resulted in 828 stems in lightly logged forests, 963 stems in medium logged forests and 1128 stems in heavily logged forests. As diversity typically increases with the number of stems (also in our study, see Fig. 2), the following diversity analyses were calculated by standardized numbers of stems, i.e. the lowest number of stems found in the plots.

**Fig. 3:**
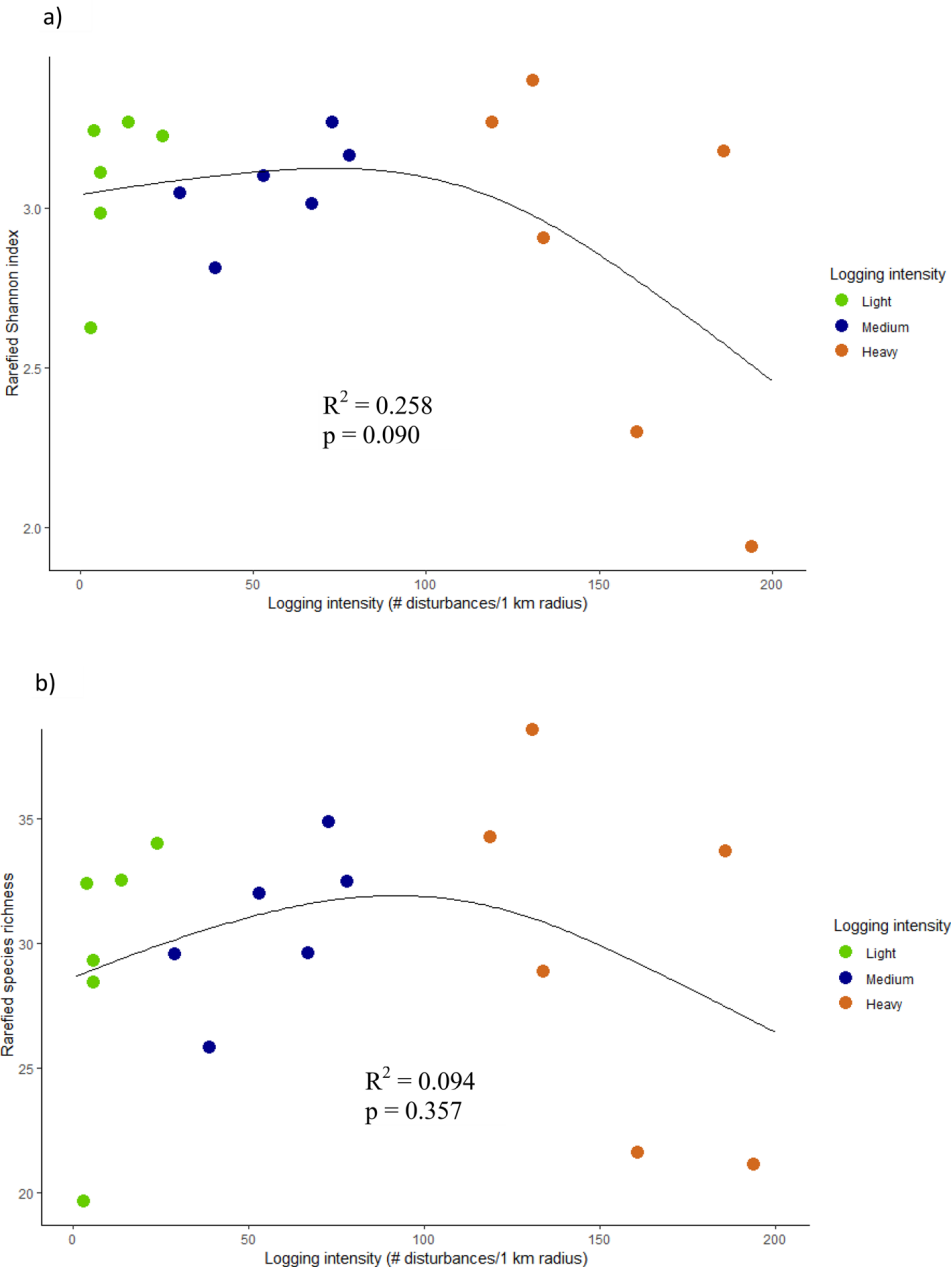
The relationship between the rarefied plot-level Shannon index (**a**) and species richness (**b**) for stems ≥ 5 cm dbh and logging intensity. Both diversity indexes have been standardized by the lowest number of stems in the 18 plots and the mean values shown are calculated based on 1000 iterations

Generalized additive models detected no significant relationships between the rarified plot-level Shannon index and logging intensity (Fig. 3a) or between the rarified species richness and logging intensity (Fig. 3b), with considerable variability in diversity among the heavily logged plots where both very high and low plot-level values were observed. When the logging intensity was classified into light, medium and heavy, we found no significant differences in diversity metrics between classes, due to the large variance in diversity metrics between plots (Table 1).

**Table 1:** Mean ± standard deviation of various diversity metrics for each logging intensity class. Values have been calculated from the mean diversity values per plot, which were standardized by the lowest number of stems *≥* 5 cm dbh in the plots and 1000 permutations (plot-level diversity metrics are shown in Supplementary Material 2)

Diversity index	Logging intensity			*p*	Test
	Light	Medium	Heavy		
Shannon	3.08 ± 0.24	3.07 ± 0.15	2.83 ± 0.59	0.465	ANOVA
Simpson	0.94 ± 0.02	0.94 ± 0.01	0.88 ± 0.11	0.200	ANOVA
Fisher’s α	15.00 ± 4.14	15.96 ± 2.82	15.68 ± 6.34	0.930	ANOVA
Species richness	29 ± 5	31 ± 3	30 ± 7	0.906	ANOVA
Evenness	0.91 ± 0.02	0.90 ± 0.02	0.84 ± 0.12	0.159	ANOVA

### Tree species composition

**Fig. 4:**
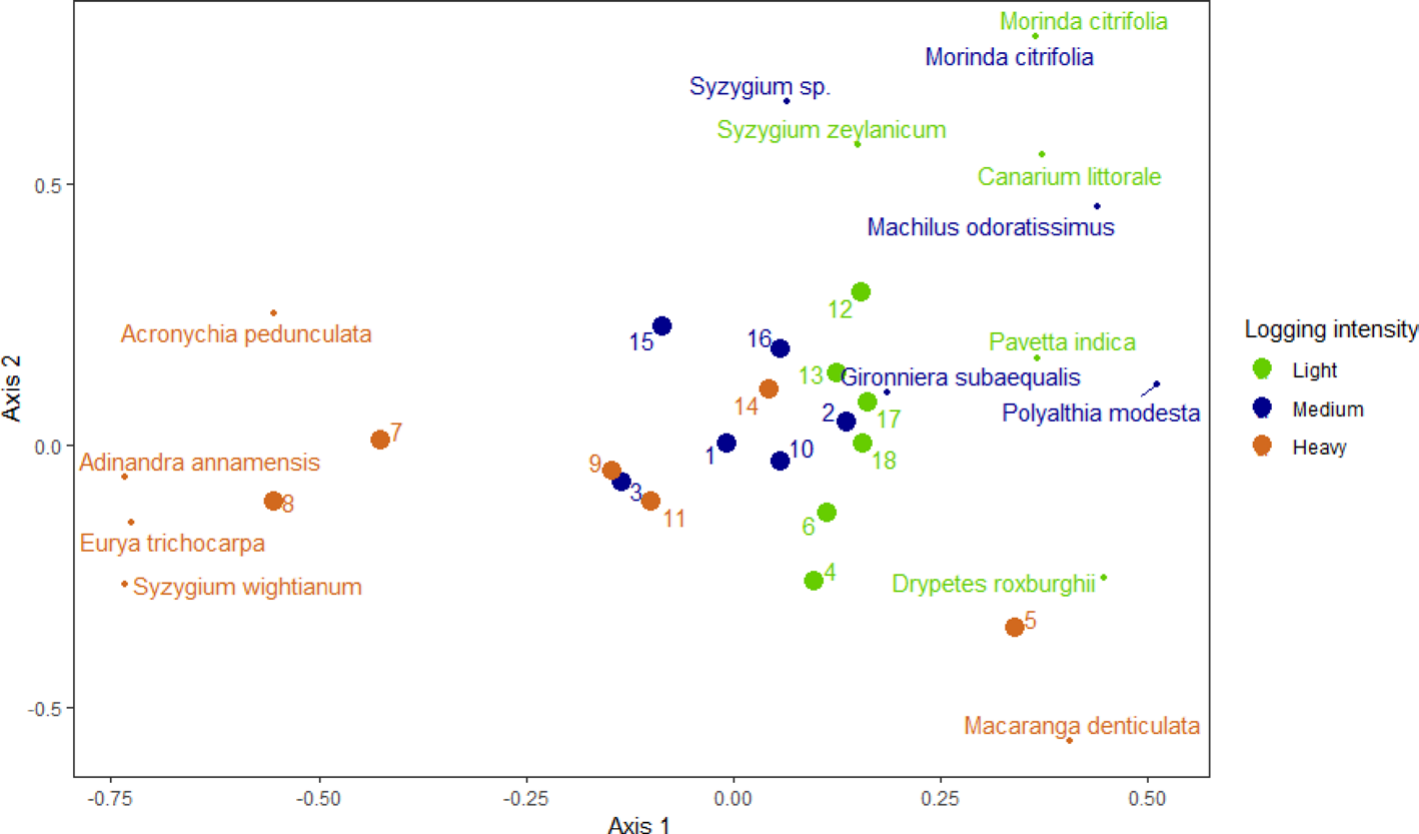
NMDS showing dissimilarity in tree species composition of stems ≥ 5 cm dbh between the 18 plots (big dots) and the five most abundant species (small dots) for each logging intensity class

The NMDS analysis (Bray-Curtis Dissimilarity) provided a good representation of the dissimilarity in tree species composition between plots, as evidenced by a low stress value of 0.1 (Fig. 4). The composition in lightly and medium logged plots was similar, while the species composition in heavily logged forests was more varied and distinct. Plots 5, 7 and 8, all heavily logged plots, had a distinct composition and also low diversity values (Supplementary Material 2). Further, deviations in forest structure were observed in these plots: plot 5 contained the lowest number of stems ≥ 5 cm dbh, i.e. 95 stems in the plot, and had a large number of banana trees. Plots 7 and 8 had the highest numbers of stems ≥ 5 cm dbh, with most stems in the dbh-class of 10–20 cm (67% for plot 7, 71% for plot 8). The total number of stems ≥ 5 cm dbh in plots 7 (251 stems) and 8 (311 stems) was considerably higher than the mean number of stems in the 18 plots, i.e. 162 stems (all plots, excluding plots 5, 7 and 8, contained between 109 and 189 stems ≥ 5 cm dbh). In general, plots located in the same geographical area were more similar in terms of species composition (see Fig. 1 for the spatial distribution of the plots).

The five most abundant species differed for each logging intensity, with only *Morinda citrifolia* being abundant in both the lightly and medium logged forests (Table 2). Four out of the five most abundant species in the heavy logging class were strongly correlated with plots 7 and 8, especially *Eurya trichocarpa* was very abundant (Table 2) and strongly determines the first axis in the NMDS plot (Fig. 4). *Macaranga denticulata*, also among the most abundant species in the heavy logging class, was frequently present in plot 5. The most abundant species in the lightly and medium logged forests were more closely grouped.

**Table 2:** The five most abundant species and their abundances for each logging intensity class. The abundances show the summation of stems ≥ 10 cm dbh in 1.5 ha and stems 5–10 cm dbh in 0.12 ha. Species are ordered by decreasing abundance per logging intensity class

Logging intensity	Species	Abundance
Light	*Pavetta indica* L.	57
Light	*Drypetes roxburghii* (Wall.) Hurus.	56
Light	*Canarium littorale* Blume	47
Light	*Morinda citrifolia* L.	40
Light	*Syzygium zeylanicum* (L.) DC.	32
Medium	*Morinda citrifolia* L.	64
Medium	*Gironniera subaequalis* Planch.	45
Medium	*Machilus odoratissimus* Nees	38
Medium	*Syzygium sp.*	37
Medium	*Polyalthia modesta* Finet & Gagnep.	36
Heavy	*Eurya trichocarpa* Korth.	206
Heavy	*Adinandra annamensis* Gagnep.	67
Heavy	*Acronychia pedunculata* (L.) Miq.	42
Heavy	*Macaranga denticulata* (Blume) Müll. Arg.	39
Heavy	*Syzygium wightianum* Wight & Arn.	39

### Wood density

Although > 80% of the stems were identified to species level, not all species were present in the Global Wood Density Database. From the 2919 stems ≥ 5 cm dbh, wood densities were assigned for 971 stems to species level, for 1647 stems to genus level and for 301 stems to plot level. The wood density of individual stems ≥ 5 cm dbh ranged from 0.310 to 0.780 g cm^− 3^ for each logging intensity class. Community wood density of stems ≥ 5 cm dbh decreased significantly with logging intensity, being 9% lower in forests with a history of heavy logging intensity than those which were lightly logged (Table 3).

**Table 3:** Mean ± standard deviation community wood density (g cm^− 3^) of stems ≥ 5 cm dbh for each logging intensity class

Logging intensity			*p*	Test
Light	Medium	Heavy		
0.574 ± 0.020^a^	0.556 ± 0.024^a^	0.523 ± 0.013^b^	0.002*	ANOVA

**Fig. 5:**
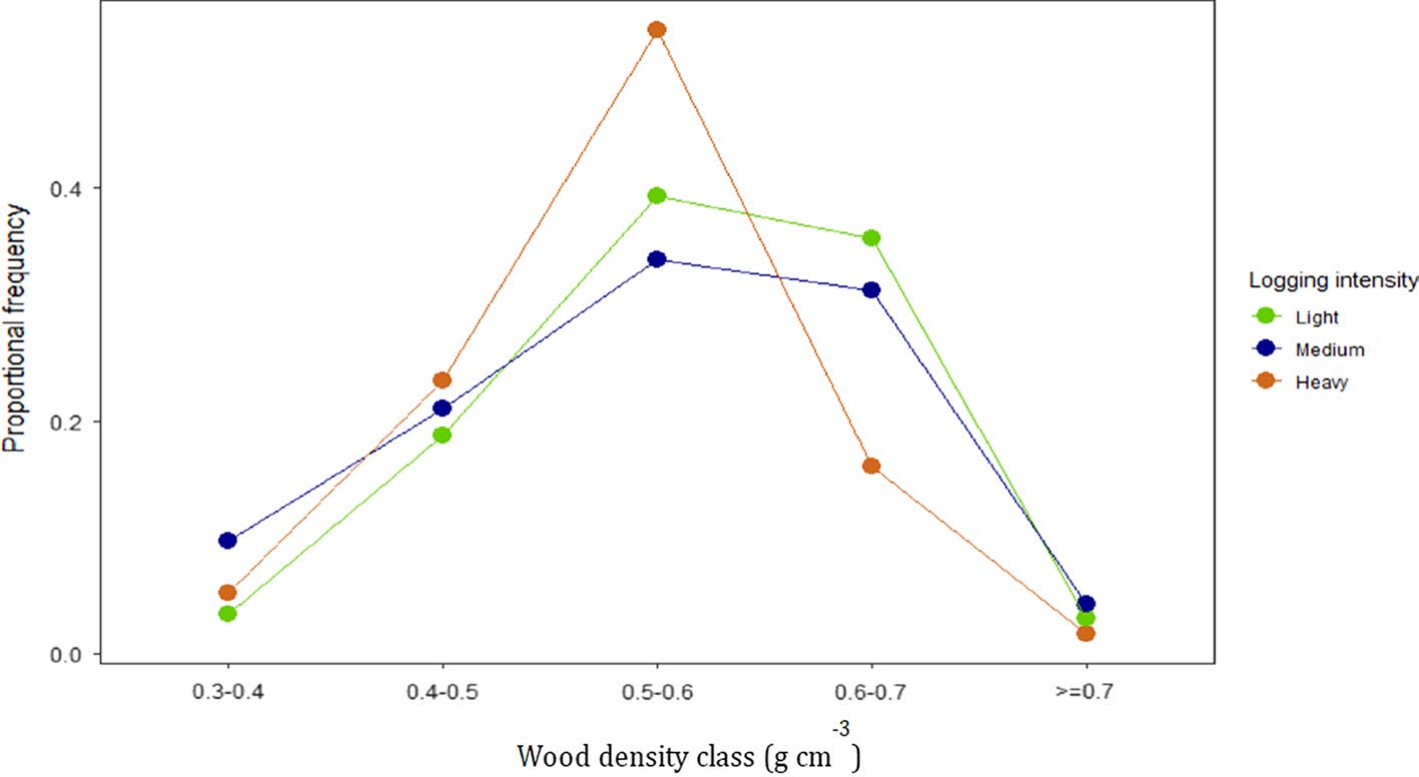
Proportional frequencies of stems ≥ 5 cm dbh per wood density class for each logging intensity class

Most stems in the lightly and medium logged forests had a wood density between 0.5 and 0.7 g cm^− 3^ (Fig. 5). In the heavily logged forests, however, 54% of the stems had a wood density between 0.5 and 0.6 g cm^− 3^, with much fewer stems with a wood density greater than 0.6 g cm^− 3^.

### Availability of timber species

Six of the 14 most extracted timber species in KNT were recorded in our plots, with only two being relatively common (Table 4). *Canarium littorale* and *Sindora tonkinensis* were more abundant and occurred in 16 and 8 plots, respectively. Only *Canarium littorale* had a decent amount of stems of harvestable size (≥ 30 cm dbh), with highest abundance in the lightly logged forests. The abundance of all timber species combined was highest in lightly and medium logged forests and most trees from harvestable size were observed in the lightly logged plots (Table 4).

**Table 4:** The abundance of timber species in the plots. The absolute number of stems ≥ 5 cm dbh of the 14 most extracted timber species in KNT (pers. comm. Viet Nature Conservation Centre, 2018) present in the plots, both below and above the minimum cutting size (30 cm dbh) and classified by logging intensity class

Species	Vietnamese name	Wood density (g cm^− 3^)	# stems 5–29.9 cm dbh			# stems ≥ 30 cm dbh		
			Light logging	Medium logging	Heavy logging	Light logging	Medium logging	Heavy logging
*Amesiodendron chinense* (Merr.) Hu	Trường	0.835	-	-	-	-	-	-
*Canarium littorale* Blume	Chủa/Trám nâu	0.495	18	25	13	29	9	7
*Chukrasia* sp.	Chua khét	0.548	-	-	1	-	-	-
*Cinnamomum ilicioides* A. Chev.	De	0.467	-	-	-	-	-	-
*Dysoxylum cauliflorum* Hiern	Dạ hương	0.715	-	-	-	-	-	-
*Erythrophleum fordii* Oliv.	Lim xanh	0.760	-	4	1	-	1	-
*Heritiera cochinchinensis* (Pierre) Kosterm.	Huỷnh	0.689	-	-	-	-	-	-
*Hopea pierrei* Hance	Kiền kiền	0.812	-	-	-	-	-	-
*Madhuca pasquieri* (Dubard) H.J. Lam	Sến	0.668	3	5	3	-	1	-
*Michelia* sp.	Giổi	0.515	4	-	1	-	-	-
*Ormosia laosensis* Niyomdham	Ràng ràng lào	0.579	-	-	-	-	-	-
*Sindora siamensis* Miq.	Gõ	0.716	-	-	-	-	-	-
*Sindora tonkinensis* K. Larsen & S.S. Larsen	Gụ lau	0.710	5	14	2	2	2	-
*Vatica cinerea* King	Táu mật	0.707	-	-	-	-	-	-
Sum all timber species			30	48	21	31	13	7

## Discussion

We assessed whether logging intensities relate to differences in tree diversity, composition, wood density and availability of timber species in KNT. Tree diversity did not differ significantly with logging intensity. The NMDS analysis showed small compositional differences between logging intensities, with each class having other abundant tree species, although the challenge of sampling at the species level sufficiently complicates interpretation. Most notably, stand-level wood density decreased markedly with increasing logging intensity and timber species were generally rare in our plots.

### The impacts of logging on tree diversity

Nine years post-logging, tree diversity did not differ significantly with logging intensity at our site. Our work suggests the likelihood of highest diversity in lightly and/or medium logged forests, but high variability in heavily logged sites means additional plots would be required to test whether patterns are consistent with the IDH. Other work supported the IDH (Connell 1978; Sheil and Burslem 2003; Bongers et al. 2009) and a meta-analysis in logged tropical forests also found that tree species richness appeared to increase at low logging intensities but decreased at higher logging intensities (Martin et al. 2015). High logging intensity generally results in a decrease in tree diversity compared to unlogged and lightly logged forests (Lindemalm and Rogers 2001; Parrotta et al. 2002).

Several Southeast Asian studies report higher tree diversity in logged than in unlogged forests, including 8 years post-logging in Borneo (Cannon et al. 1998; Sheil et al. 1999), 12–18 years in Borneo (Berry et al. 2010), and 35–40 years in China (Ding et al. 2012). The observed increase in richness after logging is likely due to an influx of generalist species that depend on disturbances and hence increase diversity metrics (Sheil et al. 1999). Differences amongst studies can be partly explained by differences in spatial scale (Sheil and Burslem 2003; Berry et al. 2008; Imai et al. 2012), logging intensity, time since logging, diversity metrics assessed, whether or not results are standardized by number of individuals and the diameter threshold considered. Because diversity metrics are scale-dependent, community similarity may provide a more robust measure of tree assemblage response to disturbance (Imai et al. 2012).

### 4.2. The impacts of logging on tree species composition

We observed a similar species composition among plots in the lightly and medium logged forests, while the composition of heavily logged sites was more distinct, especially in plots 5, 7 and 8. The most abundant species in the heavy logging class, *Eurya trichocarpa*, was very abundant in plots 7 and 8. This species usually occurs in undisturbed and slightly disturbed sites in Southeast Asia (Slik 2009). *Macaranga denticulata* was dense in plot 5 and is a light-demanding and fast-growing tree, usually occurring at forest edges or abandoned shifting cultivation lands and along road sides in Southeast Asia, which regenerates strongly in full light (FIPI 1996). After sampling the plots, local people reported that areas of forest near plots 5, 7 and 8 had been cleared and rice had been planted in the past. The remote sensing analysis showed no evidence of complete canopy removal and conversion to agriculture since 1988, therefore if any agricultural disturbances occurred this must have happened before 1988. Besides the compositional differences in these three plots, the presence of bananas, very distinct numbers of stems, high number of stems of 10–20 cm dbh and low plot-level diversity metrics further indicate that these plots have been considerably disturbed in the past and supports our understanding that plots 5, 7 and 8 contain mostly secondary vegetation. Two other abundant species in the heavily logged forest, *Acronychia pedunculata* and *Syzygium wightianum*, are light-demanding trees occurring in Southeast Asia (FIPI 1996) and are therefore associated with more open forest canopies. *Morinda citrifolia* was abundant in both the lightly and medium logged forests, but usually this species is found in open secondary forests in Southeast Asia (Slik 2009). *Canarium littorale* was among the most abundant species in the lightly logged forests and is an important timber species in KNT. This species is sporadically regenerating at forest edges and in open places and distributed across Southeast Asia (FIPI 1996). Reduced numbers of this species in the heavily and medium logged forests, especially stems from harvestable size (Table 4), is likely due to depletion by logging. Other abundant species in the lightly and medium logged forests are associated with neutral (*Syzygium zeylanicum*; shade-demanding when young, light-demanding when grown up) and high light conditions (*Machilus odoratissimus*; light-demanding, fast-growing tree that occurs in primary and secondary forests and produces a very large number of fruits and seeds) (FIPI 1996). *Gironniera subaequalis*, the second most abundant species in the medium logged forests, is a fast-growing tree that demands shade when it is young and light when it is mature and tends to develop in a dominant stand in Quang Binh Province (FIPI 1996).

Although the NMDS analysis showed that plots in the lightly and medium logged forests were grouped per logging intensity class, plots located close to each other had also a more similar species composition. Thus, observed compositional differences may reflect either natural variation in tree species or differences in logging intensity, making attribution uncertain. The challenge of sampling sufficiently at the species level in these high diversity systems complicates interpretation. A study that analysed the effects of logging on compositional diversity in tropical forests found indeed that most studies were pseudo-replicated, leading to biased estimates of change (Ramage et al. 2013).

Some studies observed a similar tree species composition in logged and unlogged tropical forests, i.e. in southern China (35–40 years since logging; Ding et al. 2012) and in the Central African Republic (6 months and 18 years post-logging; Hall et al. 2003). Others found that tree species composition differed between logged and unlogged forests in Borneo (18 years after logging; Berry et al. 2008) and between heavily and lightly logged forests in Vietnam (16 years after logging; Hoang et al. 2011). Also over time, starting just after logging and during the following 20 years, logged forests had much larger changes in tree species composition than old-growth forests in Borneo (Verburg and Van Eijk-Bos 2003). Despite a 2007 ban, selective logging continues in KNT, causing biomass losses of 3.3 Mg ha⁻¹ yr⁻¹ (Ngo et al. 2020). Ongoing extraction, together with annual typhoon disturbance (Stas et al. 2022), will further shape species composition and slow forest recovery.

### The impacts of logging on wood density and availability of timber species

We found that community wood density declined significantly with logging intensity. This is consistent with studies from Borneo and Bolivia where disturbance intensity shifted forests towards lighter-wood species (Slik et al. 2008; Carreño-Rocabado et al. 2012). This pattern likely reflects both the depletion of high-density timber species through selective logging (Table 4) and the recruitment of lower-density, disturbance-adapted taxa.

Besides stand density and tree size (dbh and tree height), wood density is a determinant of forest carbon stocks (Chave et al. 2014; Phillips et al. 2019). In Stas et al. (2020) we showed that heavily logged forests in KNT store 50% less AGC than lightly logged forests, primarily due to the loss of large (≥ 60 cm dbh) trees. Our results indicate that changes in species composition towards lighter-wood species explain roughly one-fifth (9%) of this reduction, while the remaining four-fifths reflect reduced wood volume. If logging were to cease entirely, recovery would increase carbon storage via two mechanisms: the regrowth of wood volume and large trees and a gradual shift back to higher wood density species. Together, these processes could substantially enhance both the carbon and timber value of KNT.

The 14 reported timber species (Table 4) all occur inside KNT. Only six timber species were recorded in our plots and almost all at low abundance, especially in the heavily logged forests. The absence of the remaining timber species may reflect either natural rarity or past depletion through logging. Scarcity of high-value timber species after logging was seen also elsewhere in Vietnam (Van and Cochard 2017). Silvicultural interventions such as thinning, in which the tree density is reduced to eliminate competing trees, could be applied to increase recovery of timber species and stocks (de Avila et al. 2017).

### Implications for sustainable forest management and forest conservation

Our surveys in north-central Vietnam reveal that natural forests in the region support exceptionally high tree species diversity, underscoring the importance of protecting the few remaining areas of lowland natural forest in the country. Even logged forests retained high levels of biodiversity, consistent with findings from other tropical regions (Putz et al. 2012). Notably, heavily logged forests classified as “poor” under the national system still harboured substantial tree species diversity, highlighting the conservation value of degraded forests and the risks associated with converting them to monoculture plantations. The surveyed forests are now protected as a Nature Reserve, i.e. the highest level of legal protection in Vietnam. Continued efforts to enhance forest governance and law enforcement accompanied with development of alternative livelihood strategies for forest-dependent communities remain important to cease illegal logging in KNT. Continued protection is expected to enhance AGC stocks, both through recovery of timber volume and shifts toward higher wood density species. Future monitoring will be critical to quantify rates of carbon recovery and to guide long-term conservation planning.

## Conclusions

We assessed whether logging intensity caused differences in tree diversity, composition, wood density and availability of timber species in lowland forests in Vietnam. Tree diversity did not differ significantly with logging intensity, although our work suggests the likelihood of highest diversity in lightly and/or medium logged forests. Some differences in tree species composition were observed, with each logging intensity class having distinct abundant tree species. Community wood density differed with logging intensity, with lighter wood density species in heavily logged forests. This difference in community wood density explained one-fifth of the observed difference in AGC storage between heavily and lightly logged forests. Timber species only occurred in small numbers in our plots, especially in the heavily logged forests. While logging is officially forbidden in KNT, ongoing illegal logging threatens these forests. Low logging intensities are thus important to maintain and increase community wood density, to conserve and enhance forest carbon stocks and to prevent further depletion of timber species.

## Electronic Supplementary Material

Below is the link to the electronic supplementary material.

Supplementary Material 1

## Data Availability

The data are available from the corresponding author on reasonable request.

## Code availability

Codes are available from the corresponding author on reasonable request.
