# Supplementary material for: Logging intensity alters tree species composition and wood density, but not tree diversity, in lowland forests in Vietnam
Source: Biodivers Conserv. 2026 Mar 14;35(4):110. doi: 10.1007/s10531-026-03283-2 (PMC12987793; doi:10.1007/s10531-026-03283-2)
Supplement: Supplementary file 1 — Supplementary Material 1 [file 10531_2026_3283_MOESM1_ESM.pdf]

Supplementary Information

Supplementary Material 1

Proportional frequencies of the levels of tree identifications for stems ≥ 5 cm dbh for each plot.

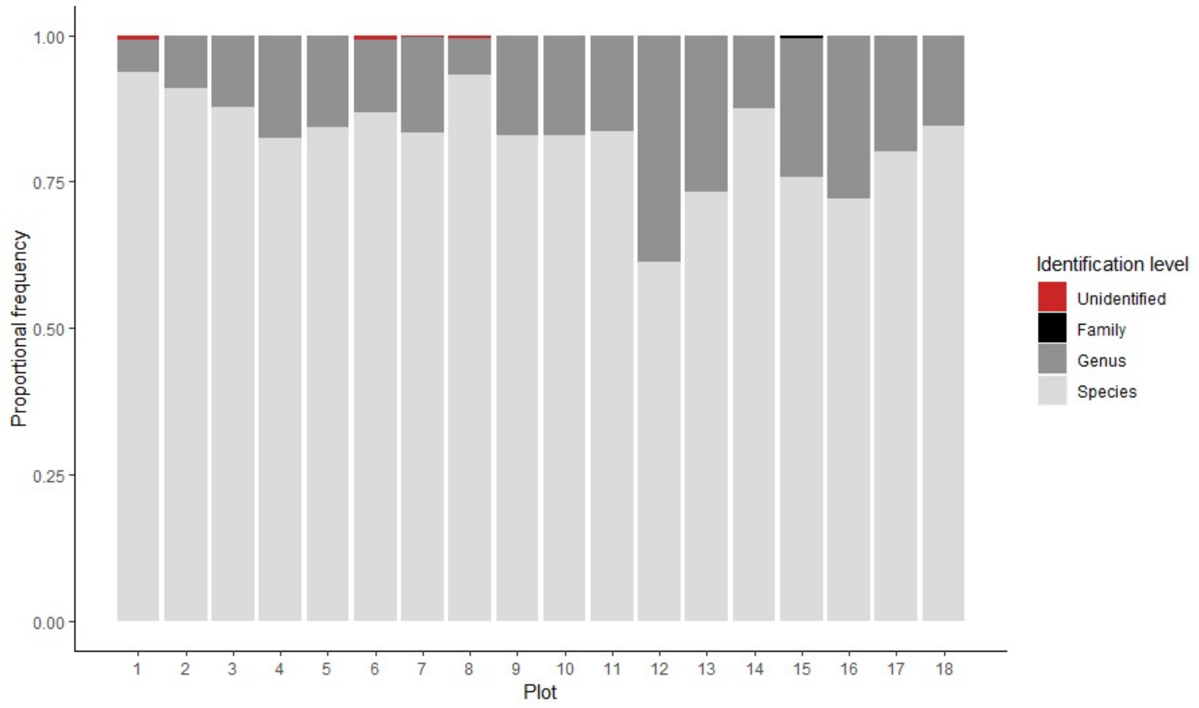

Supplementary Material 2

Diversity metrics for stems ≥ 5 cm dbh for each plot. Metrics are standardized by the lowest number of stems in the plots and permutated 1000 times to derive a mean value. Data have been ordered by logging intensity class.

Plot	Logging intensity	Shannon	Simpson	Fisher's α	Species richness	Genus richness	Family richness	Evenness
4	Light	3.27	0.95	17.53	33	27	20	0.94
6	Light	3.22	0.95	19.06	34	26	20	0.91
12	Light	2.63	0.91	7.56	20	18	15	0.88
13	Light	3.24	0.95	17.44	32	27	21	0.93
17	Light	3.11	0.94	14.57	29	24	18	0.92
18	Light	2.98	0.93	13.84	28	26	19	0.89
1	Medium	3.27	0.95	20.00	35	29	21	0.92
2	Medium	3.05	0.94	14.82	30	26	20	0.90
3	Medium	3.10	0.94	17.11	32	26	18	0.90
10	Medium	3.16	0.94	17.53	32	27	20	0.91
15	Medium	2.81	0.92	11.74	26	23	19	0.87
16	Medium	3.01	0.93	14.85	30	26	19	0.89
5	Heavy	2.30	0.80	8.76	22	20	17	0.75
7	Heavy	2.91	0.92	14.24	29	25	17	0.86
8	Heavy	1.94	0.69	8.49	21	19	15	0.64
9	Heavy	3.18	0.94	18.81	34	26	21	0.90
11	Heavy	3.40	0.96	24.41	39	31	22	0.93
14	Heavy	3.27	0.95	19.37	34	30	23	0.93
